# Understanding students’ participation in physiotherapy and nursing work settings

**DOI:** 10.1007/s10459-022-10142-6

**Published:** 2022-08-09

**Authors:** Lieke Ceelen, Anne Khaled, Loek Nieuwenhuis, Elly de Bruijn

**Affiliations:** 1 Welten Institute, Open University of the Netherlands, Heerlen, The Netherlands; 2 Research Group Vocational Education, Utrecht University of Applied Sciences, Utrecht, The Netherlands

**Keywords:** Affordances, Health profession education, Participation, Physiotherapy, Placements, Nursing, Supervising, Workplace learning

## Abstract

Students’ health profession education includes learning at the workplace through placements. For students, participating in daily work activities in interaction with supervisors, co-workers and peers is a valuable practice to learn the expertise that is needed to become a health care professional. To contribute to the understanding of HPE-students’ workplace learning, the focus of this study is to identify affordances and characterise student’s participation during placements. We applied a research design based on observations. Three student-physiotherapists and four student-nurses were shadowed during two of their placement days. A categorisation of affordances is provided, in terms of students’ participation in activities, direct interactions and indirect interactions. Students’ daily participation in placements is discussed through unique combinations and sequences of the identified affordances reflecting changing patterns over time, and differences in the degree of presence or absence of supervisors, co-workers and peers.

## Introduction

In healthcare, as in other professions, students have to be prepared for their future roles as practitioners at the workplace. Therefore, health profession education (HPE) includes placements where students are required to participate in work settings through internships, apprenticeships, and clinical experiences (Guile & Griffiths, 2001). Work settings shape students’ learning in particular ways, since learning during these placements follows from participation in daily work-related activities. In contrast to learning at school, learning at the workplace is often not intentional and not designed beforehand (Janssens et al., 2017). Little is known about students’ participation at the workplace.

To enhance HPE-students’ learning during placements, research is needed into the way students are invited to participate in work settings. The inviting quality of the workplace relates to the degree to which the workplace affords students’ participation in daily work activities and interactions (Hauer et al., 2014). Affordances are perceived as opportunities at the workplace that either already exist, or can be created in order to invite students to contribute to work and simultaneously learn at the workplace). According to Billett (2001, 2004), affordances provoke students’ participation in activities, direct interactions, and indirect interactions. Activities refer to students’ daily work actions. Direct interactions include guidance by co-workers who support students in accessing and securing vocational expertise. Indirect interactions are also important for students’ learning at the workplace, and include observation, listening and having access to specific tools and instruments.

Referring to students' participation at the workplace from a socio-cultural perspective, we perceive learning as a socially and culturally embedded process of becoming health practitioners (Engeström & Sannino, 2010; Lave & Wenger, 1991; Vygotsky, 1986). We perceive affordances as (1) situated, and (2) social in nature. Firstly, affordances are situated and cannot be dissociated from the context in which they occur. Accordingly, students’ participation in activities and interactions is embedded in particular work settings in local communities of practice (Lave & Wenger, 1991). In other words, the way work settings afford students’ workplace learning is believed to be inherently related to the characteristics of local communities of practice (Kyndt & Beausaert, 2017; Lave & Wenger, 1991). Logically, most research about students’ participation in work settings addresses specific communities of practice, such as care innovation units (Snoeren et al., 2016), skills labs (Harrison et al., 2021), hospital firms (Billett et al., 2018), or geriatric nursing homes (Goller et al., 2010; Anvik et al., 2020). However, identifying the inviting quality of different work settings in a variety of vocational communities will be helpful to improve general understanding of students’ participation at the workplace.

Secondly, since the relationship between individual learning and the environment is social in nature (Leont’ev, 1978), interactions are assumed to characterise affordances. Students gradually become participants in communities of practice through social interactions with experienced practitioners (Lave & Wenger, 1991; Vygotsky, 1986). Consequently, students are supported to participate in work activities in direct interaction with their supervisor, co-workers and peers. In this way, their learning is influenced by multiple viewpoints on practice (Morris et al., 2021). From this perspective, students’ participation includes, for example, invitations to discuss work processes actively, and engagements in guidance-oriented interactions (e.g. Gowlland, 2014; Ruoranen et al., 2017). Although prior research reveals opportunities for students’ learning, including cooperation, coaching, feedback and reflection (Kyndt et al., 2009), we want to learn more about the way HPE-students’ participation is afforded in work settings.

Affordances are believed to be situated and characterised by social participatory processes. We perceive students’ participation at work as manifestations of affordances. Consequently, the way students participate in activities, direct interactions and indirect interactions is influenced by contextualised practices of specific work settings, and shaped by ‘how we do things around here’. The rationale for undertaking this empirical study is to enhance understanding of HPE-students’ participation in placements. The research questions of this study are: (1) Which categories of affordances, in terms of students’ participation, can be identified in physiotherapy and nursing work settings? and (2) How can we characterise student-physiotherapists' and student-nurses’ daily participation in work settings through the identified categories of affordances?

## Methods

### Educational context

We performed our empirical study in placement programs of student-physiotherapists and -nurses in Dutch Universities of Applied Sciences. In the Netherlands, at Universities of Applied Sciences, HPE for physiotherapists and nurses comprises a 4-year Bachelor’s course for full-time students. During those four years, students gain clinical experience at various times. In the final year, students participate in a placement for approximately half a year. Students are paired with one supervisor at the workplace, and sometimes with two supervisors. During these final placements, the focus of student-physiotherapists’ and -nurses’ learning is predominantly on developing professional competence towards an entry-level practitioner.

### Design

A research design based on observations was chosen to enable researchers to access manifestations of affordances in placements of HPE-students. To identify categories of affordances and to describe students’ participation in work settings, seven student-physiotherapists and -nurses were shadowed during two of their placement days. Shadowing involves following participants over an extended period of time (McDonald, 2005; Vukic & Keddy, 2002). The perspective of this methodology does not seek causes or effects, but intends to provide unique insights into day-to-day workings and contextualized actions. The present study was conducted from a socio-cultural orientation, as we believe that affordances are socially embedded and situated in specific work settings. Shadowing is useful to investigate students’ participation in activities and interactions in a detailed way, as this qualitative method aims to closely follow students as they go about their daily work, with all its interacting elements such as co-workers, patient and facilities. Shadowing made it possible to capture these interplays in observations, and provide insight into manifestations of affordances taking place simultaneously. Since we aimed to investigate students’ participation in work settings authentically and contextualised, this method was appropriate.

### Participants

Using a convenience sampling method, contacts within multiple work settings were approached through the external network of key teachers in physiotherapy and nursing. These teachers, of three Universities of Applied Sciences in the Netherlands, were asked to contact workplace supervisors that they considered to facilitate representative placement experiences to students (Miles, Huberman, & Saldaña, 2014). Seven work settings participated in the present study. Table 1 represents an overview of the work settings. In each work setting one student agreed to participate in our study. The participating student-physiotherapists and student-nurses did a twenty-week placement, as part of their final year of HPE. Prior to data sampling, supervisors and students were met by the first author and informed about the ethical procedures, their voluntary participation and the research design. Participants were then given time to consider their participation, read the information letter and ask questions, before written informed consents were signed. During the moments of shadowing, verbal permission was obtained from other involved students, co-workers, and patients at workplaces.

**Table 1:** Overview of work settings

Period of data sampling	Work setting	Occupational context	Short description
February 2019 until July 2019	Rehabilitation centre (1)	Physiotherapy	Rehabilitation centre where patients often stay in-house and participate in longitudinal interprofessional treatment processes. The student-physiotherapist is paired with two supervisors. A fixed agenda determines the daily patient planning. Patient-care is provided in the common exercise room, the swimming pool, or in private rooms
	Hospital (2)	Physiotherapy	The placement includes two hospital departments: neurology and gastroenterology. Two supervisors jointly guide the student, and five other student-physiotherapists. Physiotherapists from different departments meet in the common staff room. A daily updated overview of patients indicates which of them require physiotherapeutic care today. Direct patient-care is most often provided in patient-rooms, or in public areas such as the halls and stairwell
	Private practice (3)	Physiotherapy	The private practice is located in a community health centre. The student is accompanied by two supervising physiotherapists, with various specialisations. A fixed agenda determines the daily patient planning. Patient-care is provided in private treatment rooms, and in the common exercise room. Besides direct patient-care, fitness training is provided, where people can exercise and/or rehabilitate in small groups, under professional coaching
September 2019 until January 2020	Hospital (4)	Nursing	The placement is located in the hospital department of gynaecology and urology. The student-nurse is officially paired with one supervising nurse, but also works shifts without her supervisor and with other co-workers and several other students. Each (student-)nurse has a number of own patients and carries a mobile phone; patients press a bell when they are in need of care. Direct patient care is provided in patients’ rooms, as well as during the doctor’s rounds. Handovers and consultations most often take place in the staff room
	Nursing home (elderly care) (5)	Nursing	The nursing home accommodates elderly people who can no longer live independently and require nursing care, including patients with dementia, Parkinson’s disease, physical disorders and rehabilitation. Residents have their own room, and a shared living room. The student-nurse is assigned to a supervising nurse, who works in various departments with a focus on quality assurance and education. In the daily work processes, students are most often accompanied by other co-workers, and several other peers. The treatment schedule is drawn up together on a daily basis
	Psychiatric care institution (6)	Nursing	The placement is located in a department of a psychiatric institution for people with chronic depression. Residents stay in-house and participate in longitudinal treatment processes. The student-nurse is paired with one supervisor. In addition, the small staff-team consist of a couple of other co-workers and students. Daily work processes are partly determined in a fixed agenda, and partly dependent on unpredictable patient care. The team divides the nursing tasks daily, consultations take place in the shared staff room
	Home-based care (district nursing) (7)	Nursing	Home care takes place at people’s own homes. Patients are pre-assigned among the team of nurses, each of whom walks a different route of patient visits. A certain amount of time is available per patient. The student-nurse is paired with a supervising nurse, who also has policy responsibilities in district nursing

### Data sampling

Between February 2019 and January 2020, a student in each work setting was shadowed for two days. All data was sampled by the first author. This researcher observed all their activities and interactions, including for example patient visits, feedback moments and peer discussions. An observation protocol structured the data sampling process. The observation protocol included an overview of the procedure before, during, and after the data sampling period, and a description of the needed materials, such as printed copies of research information, consent forms and the observation scheme. During the shadowing process, field notes were made in an observation scheme which was developed in line with our conceptual framework. The observation scheme distinguished four columns representing times and location in the first column, and columns for detailed descriptions of students’ participation in activities, direct interactions, and indirect interactions in the following columns (Table 2). Prior to and during data sampling, calibration on the correct completion of the observation scheme was carried out with the second author. To avoid interrupting daily work processes throughout the observation period, the researcher rarely asked questions. In exceptional case, some questions were asked for clarification, such as what was being said on the other end of a phone call. The transcripts of a day’s shadowing varied between 2100 and 5200 words.

**Table 2:** Observation scheme

Time & place	Activities	Affordances	
		Direct interactions	Indirect interactions
Start time of the activity and short description of the location/place where the activity takes place	Detailed descriptions of students’ actions in work-related activities	Descriptions, as literal as possible, of direct interactions with supervisor, co-worker or peer	Short descriptions of indirect interactions: observations or available materials, resources or tools
	What is the nature of participation in activities?	What is the nature of direct interactions?	Is there indirect access to support?
	Is there independent, in proximity, or supported participation in activities?	Who is involved in the conversation?	Who is present in the same room?

The first observation days took place between the fifth and ninth week of students’ placements. The second observation days followed at least six weeks later, and were scheduled between week thirteen and nineteen of students’ placements. Observation periods varied between 180 and 348 min, and averaged four hours and twelve minutes. References to organisations or individuals were pseudonymised in transcripts of field notes.

### Data analysis

We followed a qualitative data analysis approach as described by Miles, Huberman and Saldaña (2014). All field notes were transcribed verbatim by the first author. Initially, all authors familiarised themselves with the data by means of reading through multiple transcripts of field notes. A sub-set of data, comprising the data of four placement days, sampled in two work settings, was selected by the first author to start exploring and analysing the data (Brooks & King, 2014). In order to answer the first research question, the first and second author highlighted and discussed patterns in the field notes of students’ participation in activities, direct interactions and indirect interactions which might potentially contribute to the identification of categories of affordances in this sub-set of data. The first author open-coded the transcripts line by line, following a group meeting to discuss and define preliminary categories. Subsequently, all transcripts were coded in the preliminary categories of affordances by the first author. To ensure reliability of coding, fragments were partly coded independently by the first and second author, followed by consensus meetings with all authors in which similarities and differences were discussed. In these meetings, the preliminary categories were evaluated by abstraction, and further reduced. Inductive saturation appeared when no new categories were identified in the data (Saunders et al., 2018). This process resulted in the identification of five categories of affordances reflecting students’ participation in physiotherapy and nursing work settings.

To characterise students’ daily participation in work settings through the identified affordances, the first author reorganized the textual transcripts by creating matrices (Miles et al., 2014). In these matrices, for each placement day, students’ participation in activities, direct interactions and indirect interactions were highlighted in five different colours, representing the categories of affordances (Table 3). In this way, manifestations of affordances in students’ daily participation in work settings were visualised. In meetings with all authors, differences and patterns of students’ participation in work settings were described using a constant comparison approach to systematically discuss similarities and differences between placement days, within and across work settings (Glaser & Strauss, 1967; Lincoln & Guba, 1985). To externally validate the identified categories of affordances and to discuss students’ daily participation in work settings through the identified categories, results were presented by the first author in academic peer meetings and in conference sessions, including the online EARLI 2021 conference.

**Table 3:** Affordances in physiotherapy and nursing work settings

Categories of affordances	Students’ participation in placements		
	Activities	Direct interactions	Indirect interactions
Interaction before and after caregiving activities	Active participation in interactions, before and after caregiving -Interaction about day-planning, preparing or dividing work activities -Discussing patient cases and receiving feedback on caregiving activities -Reflective conversation about learning progress, personal development, and views on professional practice	Interactions before and after providing direct patient care -Organising and influencing the scheduling of work activities -Making clinical reasoning explicit, being questioned about patients' cases and work approach, receiving feedback on provided patient care, and engaging in short discussions aimed at providing brief feedback on caregiving -Being listened to, being encouraged to reflect and explicate views on professional practice, and being challenged to make learning objectives and challenges explicit	Presence of supervisor, co-worker, or peer -Available time for interactions, before and after providing direct patient care -Staff- or private rooms for calm conversations
Observing caregiving activites	Passive participation in caregiving activities, observing others’ caregiving activities	Limited interactions -Receiving explanations and getting involved in patient-interactions	Presence of supervisor, co-worker, or peer -Observe provided care -Listening to supervisors, co-workers, or peerswho talk out loud and interactions with patients
Providing care with direct support	Supported active participation in caregiving activities -Receiving and processing direct support while providing patient care -Updating and adjusting caregiving activities	Direct supportive interactions, talks between an expert and a novice -Receiving direct verbal support while providing patient care: feedback, questions, explanations and suggestions while providing patient care -Receiving physical support while providing patient care	Presence of supervisor, co-worker, or peer -Supervisor, co-worker or peer who also monitors and responds to patients' feedback -Supervisor, co-worker or peer who keeps track of time
Providing care in proximity	Active participation in caregiving activities -Preparing, providing, and administering patient care -Interacting with patients (formal and informal conversation) -Processing patients' feedback	Peer-to-peer interaction, discussions as reasonable equal colleagues, or no interactions -Talking out loud and discussing work-approach while providing care -Talking about division of work activities -Requesting or receiving confirmation or feedback for small adjustments (verbally or nonverbally) e.g. nodding affirmatively, small suggestions, tips or hints	Presence of supervisor, co-worker, or peer -Being observed and monitored
Working individually	Active participation in caregiving activities -Preparing, providing, and administering patient care independently -Interacting with patients (formal and informal conversation) -Processing patients' feedback	No direct interactions with supervisor, co-worker or peer	Absence of others -Supervisor, co-workers, or peers are contactable, altough not present -Schedule, patient-planning and division of work activities -Patient information at (mobile) computers, printed overviews, phone, or hard copy folders

## Results

The data revealed five categories of workplace affordances: (1) interaction before and after caregiving activities, (2) observing caregiving, (3) providing care with direct support, (4) providing care in proximity, and (5) working individually. The specifications of affordances found in fourteen placement days, in seven work settings, are summarized in Table 3. Firstly, we address each of the categories of affordances separately. Secondly, we characterise students’ daily participation in work settings by means of the identified categories, revealing sequences and combinations of affordances throughout students’ placement days.

### Affordances identified in the context of physiotherapy and nursing students’ placements

#### Interaction before and after caregiving activities

During their placements students were able to discuss work and learning activities, before and after direct patient care. The topics of conversation varied. Some shorter examples of work-interaction included concise feedback moments or brief patient discussions. These discussions took place in consultation rooms, but also at the coffee machine, or on the way to the next patient. In other discussions, supervisors explicitly discussed students’ learning activities and difficulties. Learning progress and professional reasoning were subjects of these conversations.

In the rehabilitation centre (1), the student-physiotherapist and her supervisor start their workday together at eight am. With the first patient arriving at half past eight, they take time to talk about the upcoming work day together. They discuss the patients, and divide work activities. (..). The nature of the conversation changes when the supervisor asks her student if there are any further things she wants to go through. The student-physiotherapist tells her supervisor how she struggles with her views on professional practice. In her previous placement, things were different. She has the feeling that she now has to ‘unlearn’ things that she had just learned half a year ago at her previous placement. She gives an example of how she previously learned to approach treatments of lower back pain differently. Her supervisor explains how these different approaches are not either good or bad, when the rationale for practice is right. The student explains how she struggles to structure and justify her reasoning in order to provide evidence-based practice.

#### Observing caregiving activities

Students were allowed to observe the caregiving of their supervisor and other co-workers. In this way, students were present at the scene, but played no active role in work activities. While observing others’ caregiving activities, students were seen to be more or less included in direct interactions with patients.

In the hospital (4), the student-nurse and her supervisor visit a patient together. (..) The supervisor provides medicines and explains out loud what the medication does, and how she scans and administers it. Next, the supervising nurse replaces the patient’s stoma while the student-nurse observes her. Occasionally, she asks the student-nurse to indicate materials, and she explains and instructs the steps for stoma care out loud.

#### Providing care with direct support

During their placements, students were entrusted to participate actively in patient care activities with direct supervisor support. Supervisors were able to guide students’ work activities by describing the next step, asking questions, giving explanations, or providing physical support.

In the hospital (2), the student-physiotherapist is asked questions while providing patient care. When the patient bends and extends his leg, the supervising physiotherapist asks the student whether this leg is in full extension. When the student-physiotherapist remains quiet, she continues by asking how they could extend the leg even further. The student indirectly answers her by explaining to the patient how to practice the leg’s extension by pressing the knee as far as possible into the bed. The students’ supervisor nods and suggests to measure the patients’ extension. The student-physiotherapist takes the measurements and writes down the values.

In some situations, supervisors tried to steer students’ actions verbally. In the example below, the supervisor’s verbal support was followed by a change of role. At a certain point, the student literally steps back when the supervisor takes over from her by adjusting the patients’ exercise.

In the private practice (3), the supervising physiotherapist stands alongside the student when the patient asks the student if she can do abdominal exercises. The supervisor mentions to the student-physiotherapist to take into account that the patient cannot easily get to and from the ground because of her operated knee. The supervising physiotherapists further says aloud that abdominal exercises can be done standing up, or, for example, from bed. Next, the student-physiotherapist explains an exercise, and encourages the patient to practice this exercise on the steps. (..) The supervisor observes this and asks the student what muscles the patient specifically is training in this exercise. The student answers (..), to which the supervisor responds: “I am sorry, but I am going to adjust this exercise”. The student listens while her supervisor explains another exercise to the patient.

#### Providing care in proximity

While providing care in proximity, there was not always a clear distinction between the role of the experienced colleague and the student as beginner. This included situations where students work independently, while others are present.

In the psychiatric care setting (6), the student-nurse, her supervising nurse and a physician together attend a meeting in the morning with patients which is named the day-start. The student-nurse asks the first patient to start. The patient talks about the activities she will undertake today. Then, the other patients follow. The student-nurse asks each patient some follow-up questions such as “What do you think of your treatment schedule?”, “Did you include enough moments of rest?”, or “Have you thought about some exercise?”. Once, her supervising nurse complements the student by advising a particular patient to go and see a specific activity.

Collaborative work in patient care was also found in this category of affordances. Specific work tasks were then divided between the supervisor and the student.

In the hospital (4), the student-nurse and her supervisor take care of a patient together. While the student-nurse replaces the patient’s bag of urine, her supervising nurse is changing the patient’s bed.

#### Working individually

Students were invited to participate in patient care individually. Affordances in this category include students’ individual preparation or administration of patient care activities.

In the home-based care setting (7), the student-nurse is challenged to work individually, without direct interaction with her supervisor, co-workers or peers. The work schedule on her mobile phone provides a structure for her patient visits. By bicycle or on foot, the student-nurse moves from home to home to provide patient care independently. At patients’ homes, this student-nurse is facilitated to work with patient folders, including written patient information and overviews of caregiving tasks.

### Characterisation of students’ placement days in work settings

**Fig. 1:**
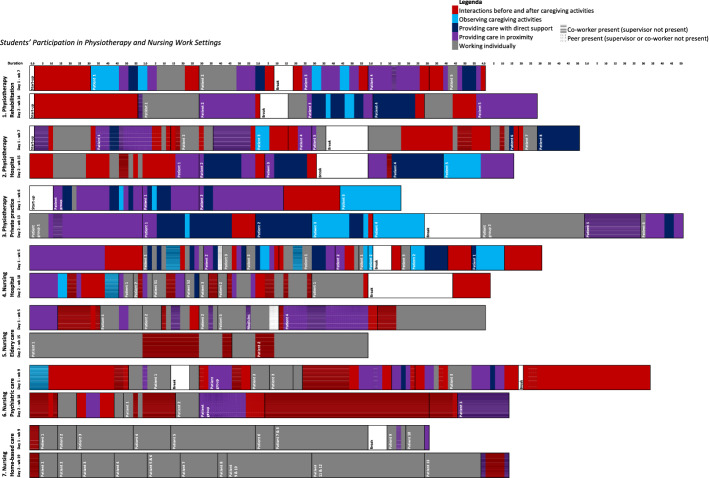
Students’ participation in physiotherapy and nursing work settings

In the seven placements, students are stimulated to learn through unique combinations and sequences of the identified affordances (Fig. 1). Students’ daily participation in placements could be characterised through changing patterns over time, and differences in the degree of presence or absence of supervisors, co-workers and peers.

#### Uniqueness of work settings

Each work setting reveals its own pattern of affordances. In some work settings, students work for longer periods of time within particular categories of affordances, in others, several categories alternate more quickly for multiple shorter periods in a row. Accompanying Fig. 1, below, a brief outline is given per work setting.

In the rehabilitation centre (1) both placement days start with interactions between the supervisor and student-physiotherapist about work, and the student's learning progress. The interactive start is followed by several patient treatments. The categories of affordances alternate per patient: provide care with direct support, in proximity, or individually. Occasionally the student observes her supervising physiotherapist providing patient care. The patient visits are interspersed with short feedback moments. Both on the first and second day, there is one short moment where the student’s work interacts with another more experienced co-worker (other than her supervisor).

In the hospital setting (2), both the physiotherapists’ work activities and the categories of affordances alternate more quickly than in the private practice. The student-physiotherapist prepares patient visits and administers patient data mostly individually. Patient visits usually take place in the presence of peers, co-workers, or supervisor. At a number of times, direct support is provided by the supervisor, and once, direct support is provided by a peer. Almost all patient cases are discussed with the supervisor before and/or after patient visits. The student not only discusses patient cases with his supervisor, but also with other co-workers. On both days, the student observes a patient treatment by his supervisor once.

In the private practice (3) the supervisor literally stands beside the student-physiotherapist for most of the time, both days. However, on the second day of observation, the student was seen to provide a group lesson individually, without the presence of her supervisor. Work activities and categories of affordances alternate more discretely in this work setting. The proximity of the supervisor allows her to provide just-in-time guidance, and also, to take over the student's work on a number of occasions. The student observes several patient treatments provided by her supervisor. Most direct interactions between supervisor and student take place during patient care activities. Unlike in the previous work settings, there are only a few moments for interaction before and after caregiving activities.

In the hospital setting (4), the student-nurse is confronted with frequently changing categories of affordances. Work activities with and without direct support often alternate, and patients’ caregiving activities are regularly varied with short interactions about work. The student-nurse’s work activities are interrupted multiple times, for example by requests from other patients, co-workers or the doctor’s visit. Interactions, before and after caregiving activities, are frequent and usually brief. On the first day, demonstrations of work activities and direct support were provided by the supervisor at a number of brief moments. On the second day of observation, direct support was only provided once. The student works mostly independently and engages in multiple conversations with co-workers, other than her supervisor.

In the nursing home (elderly care) (5), the student-nurse visits patients individually, or in the presence of another co-worker or a peer student. Unlike in most other work settings, the supervisor is only present at the start of the day, and only comes back once during the day to shortly monitor how things are going. During the second observation moment, the student does not see her supervisor. Although the student often works in close proximity to another, the conversations between the student and colleagues do not have the character of direct support. The topics of conversation, before and after caregiving, are usually practical in nature and concern, for example, patient division and planning. On the second day of observation, the student visits patients independently. In between, the student briefly discusses a patient case with colleagues.

In the psychiatric care institution (6), a relatively large number of moments to discuss patient cases with colleagues is observed. These interactions, before and after direct patient contact, take place with several co-workers, and students. Unlike in the other work settings, relatively little direct contact is seen with patients. The student-nurse meets patients both individually, for example to hand out medicines, and in groups. Patient group meetings take place in the presence of her supervisor, co-workers and/or peer-students. The students’ supervisor hardly provides any direct support in the presence of patients. There are, however, multiple feedback meetings before and after patient contact.

In home-based care (district nursing) (7) the student-nurse works mostly independently. She spends most of her placement day alone, with patients, which is visualised by a practically unambiguous pattern of the category ‘work and provide care individually’. During both days, there is no direct interaction with her supervisor. Once, the student calls a colleague to ask for permission to give medication to a patient. At the beginning and end of the shift, there is brief consultation with other co-workers.

#### Patterns of affordances over time

When comparing the data sampled at the beginning and at the end of students’ placements, in four work settings an increase was shown in moments of working individually. Students were afforded more often to work individually on the second day of data sampling, compared to the first, in the rehabilitation centre (1), the private practice (3), the hospital (4), and in the nursing home (eldery care) (5). The degree of individual work in home care (7) is high on both of the student’s placement days. In the hospital (2) and the psychiatric care institution (6) we did not find an increase in the duration of the category ‘work individually’.

In the private practice (3), the student-physiotherapist accompanies patient group lessons both on the first day and the second day of data sampling. On the first day her supervisor remains present. However, on the second observation day, later in the term, the student-physiotherapist was seen accompanying a similar patient group lesson independently and individually.

It is noticeable that in all physiotherapy work settings (1, 2 and 3), the category ‘provide care with direct support’ increases on the second day of observation, compared to the first day of observation. In the nursing work settings (4, 5, 6, and 7), we found the opposite: the category ‘provide care with direct support’ decreases on the second day of observation, compared to the first, or remains low.

The student-nurse’s work activities in the hospital (4) are interrupted for direct supervisor support more often on the first day of data sampling than on the second moment. Similarly, in elderly care (5), the student-nurse’s moments of individual working and caregiving become longer on the second moment of data sampling.

#### Presence of supervisors, co-workers and peers

Our data reveals differences in the presence of others during the observed placement days. We have found students who work in pairs with their supervisor, students who work with co-workers or peers, and students who hardly see any other colleagues at all. In some work settings, several colleagues work in the same work space, making proximity to others more of a standard practice.

In the rehabilitation centre (1), the student-physiotherapist provides patient care independently, while her supervisor and other co-workers are present at the same time in the spacious exercise room. While working in each other’s proximity, her supervisor is able to keep an eye on her, and parallelly treat another patient in the same exercise room.

Most students participated in work processes and interactions in the presence of others. Exceptions are the high degree of individuality found on the second day of observation of the student-nurse working in elderly care (5), and the student-nurse working in home-based care (7). The data also shows that the student-physiotherapists work more often in close vicinity of their supervisors than the student-nurses. Particularly the work of student-nurses in the nursing home (5) and the psychiatric care institution (6) interfaces at several times with various co-workers.

In the elderly care setting (5), the student-nurse and a peer-nurse briefly reflect on the joint caregiving activity they have just undertaken. They washed and dressed a lady together. When they are in the hall again, they talk about what went well, and what could be improved. The student-nurse explicates that, to improve their procedure for washing the lady, they should observe how a more experienced colleague takes care of this lady. The peers agree to ask a co-worker for help later today, and they continue to the next patient room.

In four out of seven work settings, students were found to interact with student-peers. The nature of the interactions between peers differed. In the nursing home (5), students were mostly observed to work together, as equal colleagues. In the hospitals (2 and 4), the students' caregiving activities seemed to interface less, but students knew where to find each other for short consultations or social talk. In the psychiatric care institution (6), we observed that the (senior) student-nurse takes a (junior) student with her a number of times throughout the second observed placement day.

In the psychiatric care institution (6), the student-nurse meets a patient to discuss the treatment plan. On her way with the patient to find a consultation room, the student walks past the staff room. She sees the junior-student sitting there, and asks him if he would like to join the patient's treatment plan discussion with her. The junior-student looks at his supervising colleague. "Good idea" says the colleague, "it is important to see as much as possible during your placement”. The junior-student goes along, and listens in on the treatment plan discussion.

## Discussions

Our most important finding is the categorisation of affordances, which allows us to characterise HPE-students’ participation at the workplace. In general, the findings indicate that work settings invite students to gain meaningful learning experiences in their placements, as a result of active participation in daily work processes at work. The chosen research method has been appropriate for investigating affordances in terms of students’ participation. Following a contextualised perspective on affordances, we found that work settings are distinctive in the ways they stimulate students’ workplace learning and that each placement day is shaped by unique combinations and sequences of these categories.

The five categories of affordances enable the understanding of the inviting qualities of work settings and the opportunities to stimulate HPE-students’ learning. Generalisations of our findings are difficult to apply because we investigated unique learning situations in seven different work settings. Nevertheless, we recognise that our findings are in line with previous research. This enables us to discuss the tentative relationship between the categories of affordances and students’ workplace learning. The category ‘work individually’ involves students’ independent practice and could be related to the concept of entrustment. Entrustment involves decisions to let the student participate autonomously in work activities (Ten Cate et al., 2013). Independent practice is commonly considered important, because it is believed to contribute to the development of students’ skills, confidence and professional identities (Bremer et al., 2022; Snoeren et al., 2016). ‘Provide care in proximity’ could afford students to learn safely through trial and error. Supervisors or other co-workers are present to provide help when needed and monitor students’ level of understanding (De Bruijn, 2012; De Vos et al., 2019; Harteis & Bauer, 2014; Van der Leeuw et al., 2018). ‘Provide care with direct support’ might illustrate the supportive culture of work settings, in which students can learn through supported participation and are afforded opportunities to receive direct feedback (Billett, 2004, 2016)*.* Affordances to ‘observe caregiving activities’ show how students are invited to participate through observations in placements. This involves experts’ demonstrations of vocational knowledge and skills, including the out-loud articulation of their clinical reasoning (Ceelen et al., 2021*).* Besides the varied possibilities for engaging in caregiving activities, most students were invited to participate in all kinds of social interactions. Affordances to ‘interact before and after caregiving activities’ could, for example, include joint reflections of the performances of the student (Billett et al., 2014). Although the five categories were briefly approached separately, we assume that the learning potential could best be found in the unique combinations of workplace affordances.

The combinations in which the distinctive categories represent daily participation in placements reveal interesting configurations of affordances. For example, scaffolding strategies can only be seen in a series of sequential categories involving the students’ activity to be monitored (‘provide care in proximity’), steered through explanations (‘provide care with direct support’) and demonstrated through taking over parts of the activity that the student is not yet able to do (‘observe caregiving’). However, when analysing students’ daily participation in work settings, it was difficult to find such typical structures. We hardly ever observed that the categories of affordances followed comparable patterns. Instead, the combinations and sequences of affordances appeared to be unique throughout all placement days (Fig. 1). Nevertheless, the findings indicate some interesting insights regarding the characteristics of work settings, the presence (or absence) of others, and changes of affordances over time.

The unique configurations of affordances show how student-physiotherapists and -nurses are stimulated to learn differently, depending on the work setting in which they are placed. Different sequences and alternations of affordances could possibly be explained by the nature and traditions of professions, and the values and norms in specific work settings (Morris et al., 2021). For example, it could be in line with a historical tradition for nurses working in home-based care to work more individually than, for instance, nurses working in hospitals or psychiatric care. Alongside historical reasons, the focus on independent work of student-nurses in home-based care is obviously related to financial, societal and demographic reasons. In line with previous research (Goller et al., 2019), we have the feeling that experienced nurses in home-based care may have deep-rooted values to prepare students in such a way that students contribute as new team members fully to the work in the shortest time frame possible. Furthermore, whereas nurses may have traditionally worked in teams, in physiotherapy there seems to be a centuries-old tradition of individual novices who learned the profession at the side of a master. That may partly explain why we found that the student-physiotherapists worked more often in the immediate vicinity of their daily supervisor, compared to the student-nurses. However, explanatory statements are not appropriate to the contextualised approach of our study and there are more reasons, such as pedagogic reasons, that determine how students are invited to learn in work settings.

As stated in the introduction, interactions with others are assumed to positively influence students’ workplace learning. In the seven work settings, most students participate at work in close company of their supervisors. This seems a relevant finding since the presence of supervisors, and other co-workers, could be related to providing a safe learning environment for students in which direct support and interactive participation stimulates students to grow professionally and develop work-strategies (Harteis & Bauer, 2014; Van der Leeuw et al., 2018). Indeed, the affordances may indicate how the presence of a supervisor could result in supported participation and just-in-time interventions (e.g. Billett et al., 2014; Ruoranen et al., 2017). However, in some work settings, the students’ placement seems to be foremost a work environment, with less opportunities to learn in the presence of a supervisor. In these work settings, students had limited access to direct interactions which might imply sub-optimal learning. Nevertheless, not only the interactions with supervisors, co-workers and peers are believed to afford students’ learning; the caregiving activities, including contact with patients, could presumably function as feedback on their own (Bremer et al., 2022).

We had expected to find an increase in the autonomy of students’ participation at work over time. Indeed, in some work settings, we found that affordances regarding individual work increased, and affordances regarding direct support decreased. Nevertheless, in other work settings, especially in the physiotherapy work settings, supervisors seemed to be continuously present alongside the student, also towards the end of their placement. The categories of affordances do not reveal supervisors’ pedagogic perspective and reasons why they decided to entrust students to work individually, or not to do so. Supervisors are challenged to ensure accessibility, affordability, and quality of care, whilst offering safe learning conditions and supported participation for students (Billett, 2016; Dornan et al., 2007; Verhees et al., 2021). Since supervisors are known to be continuously challenged with considerations of students’ needs and readiness (De Vos et al., 2019; Ten Cate, 2013), it would be interesting to further review supervisors’ strategies, motives and conceptions (De Bruijn, 2012; Khaled et al., 2021). Insights into interrelated processes of supervisors' actions and reasons will enable us to further explore students’ participation at work, and the changes over time, from a pedagogic perspective.

Lastly, it is not our aim to relate affordances to the students’ actual workplace learning. As we know, this depends not only on the inviting qualities of the workplace, but certainly also on the individual student’s choices to elect or refuse to participate in affordances provided by the workplace (Bryson et al., 2006). The reciprocal interplay between affordances and the students’ agency encourages students’ learning and professional development (Billett, 2001; Goller et al., 2019). For example, proactive students could have created meaningful learning opportunities through the agentic seeking of information and feedback, even in environments where direct interactions seemed to be limited. Future research into students' perspectives is recommended to understand how students’ agency interacts with the inviting quality of work settings.

## Limitations

This study has some limitations. (1) The first limitation that is encountered in shadowing, as a qualitative research technique, is the possible effect that a researcher has on the situation they are researching. Although the first author did not feel she actually interrupted the normal work activities, the participants noticed her presence which may have influenced their work of activities. The possible observer effect was discussed with the participants after the observation (McDonald, 2005), and included questions about how ‘normal’ their day has been while the researcher was present. In order to comfort the participants, the researcher indicated beforehand that the research was descriptive in nature, and did not attempt to judge whether learning and supervision are ‘good’ or not. (2) A second limitation may be that generalisations of our findings should be formulated very carefully since we conducted present study from a contextualised perspective. Investigating workplace affordances in the context of seven unique work settings is considered as robust in qualitative research (Poortman & Schildkamp, 2012), but does not allow us to make normative statements about, for example, the quality of workplace learning and supervision. This brings us to a third limitation. (3) The observations provided us with a rich data set, but did not give us detailed insight into the reasoning of participants. We do expect the reasoning of supervisors and students to have an impact on the affordances provided in work settings. For example, from the supervisor's perspective, their perception of the student’s performance may impact the affordances provided. Including interviews with supervisors will provide more in-depth information regarding supervisors’ decisions and reasoning whether to, for example, provide direct support or entrust work activities to students. Similarly, HPE-students’ active engagement in activities and interactions is required for meaningful learning to take place. Since students elect how they engage with what is afforded to them, research on the reasoning of students will lead to more robust findings on how they give meaning to participatory practices.

## Implications

The presented insights into students’ participation in work settings extend current knowledge about the facilitation of students’ workplace learning. Careful attention to the ways students are invited to engage in work activities and interactions can have a positive effect on the professional development of students. Those involved in internship programs may do well to consider the variety of opportunities for students to participate in work settings.

Practically, this study might serve as a hold for supervisors and educators when considering and discussing the possible variations in guiding students at work. We have shown that supervisors contribute in their own unique ways to students’ participation in daily work activities and interactions. While some supervisors prefer to observe most students’ actions themselves, other supervisors entrust students to work mostly independently. Supervisors’ awareness of their guidance practices with regard to the inviting qualities of the work setting can positively contribute to students’ workplace learning. With regard to training HPE-students in clinical settings, specific guidance about the different possibilities to afford students’ participation at work is recommended.

## Conclusions

Workplace learning becomes increasingly important to prepare HPE-students for their future professions. We were able to shed some light on how students’ participation is stimulated in work settings. This study reveals an empirically-grounded categorisation of affordances which enabled us to discuss unique configurations in which physiotherapy and nursing students participate in work settings. In these configurations of students’ participation, we found differences over time and between work settings, including the degree of proximity to supervisors, the degree of interaction possibilities, and the degree to which students work individually. The study shows how each work setting affords unique participatory practices for students. The daily participation of some students involved frequently varying sequences of affordances, whereas other students’ participation could be characterised as being more monotonous. As this study only identified and described affordances in placements, more in-depth research into the relational dynamics between affordances and individual engagements will give insights into how to maximise learning opportunities in work settings.
